# Affect during incremental exercise: The role of inhibitory cognition, autonomic cardiac function, and cerebral oxygenation

**DOI:** 10.1371/journal.pone.0186926

**Published:** 2017-11-01

**Authors:** Weslley Quirino Alves da Silva, Eduardo Bodnariuc Fontes, Rodrigo Menezes Forti, Zayonara Larissa Lima, Daniel Gomes da Silva Machado, Andréa Camaz Deslandes, Erika Hussey, Nathan Ward, Rickson Coelho Mesquita, Alexandre Hideki Okano, Hassan Mohamed Elsangedy

**Affiliations:** 1 Department of Physical Education, Federal University of Rio Grande do Norte, Natal, RN, Brazil; 2 Graduation Program on Physical Education, Natalense Faculty of Education and Culture, Natal, RN, Brazil; 3 Institute of Physics “Gleb Wataghin”, University of Campinas, Campinas, SP, Brazil; 4 Brazilian Institute of Neuroscience and Neurotechnology (BRAINN), Campinas, SP, Brazil; 5 Center of Physical Education and Sport, Londrina State University, Londrina, PR, Brazil; 6 Institute of Physical Education and Sport, State University of Rio de Janeiro, Rio de Janeiro, RJ, Brazil; 7 Center for Applied Brain and Cognitive Sciences, Tufts University, Medford, MA, United States of America; 8 Cognitive Science Team, Natick Soldier Research, Development, and Engineering Center, Natick, MA, United States of America; 9 Department of Psychology, Tufts University, Medford, MA, United States of America; Radboud Universiteit, NETHERLANDS

## Abstract

**Background:**

Pleasure is a key factor for physical activity behavior in sedentary individuals. Inhibitory cognitive control may play an important role in pleasure perception while exercising, especially at high intensities. In addition, separate work suggests that autonomic regulation and cerebral hemodynamics influence the affective and cognitive responses during exercise.

**Purpose:**

We investigated the effects of exercise intensity on affect, inhibitory control, cardiac autonomic function, and prefrontal cortex (PFC) oxygenation.

**Methods:**

Thirty-seven sedentary young adults performed two experimental conditions (exercise and control) in separate sessions in a repeated-measures design. In the exercise condition, participants performed a maximum graded exercise test on a cycle ergometer as we continuously measured oxygen consumption, heart rate variability (HRV), and PFC oxygenation. At each of 8 intensity levels we also measured inhibitory control (Stroop test), associative and dissociative thoughts (ADT), and affective/pleasure ratings. In the control condition, participants sat motionless on a cycle ergometer without active pedaling, and we collected the same measures at the same points in time as the exercise condition. We evaluated the main effects and interactions of exercise condition and intensity level for each measure using two-way repeated measures ANOVAs. Additionally, we evaluated the relationship between affect and inhibitory control, ADT, HRV, and PFC oxygenation using Pearson’s correlation coefficients.

**Results:**

For exercise intensities below and at the ventilatory threshold (VT), participants reported feeling neutral, with preservation of inhibitory control, while intensities above the VT were associated with displeasure (*p*<0.001), decreased inhibitory control and HRV (*p*<0.001), and increased PFC oxygenation (*p*<0.001). At the highest exercise intensity, pleasure was correlated with the low-frequency index of HRV (r = -0.34; p<0.05) and the low-frequency/high-frequency HRV ratio (r = -0.33; p<0.05). PFC deoxyhemoglobin was correlated with pleasure two stages above the VT (r = -0.37; p<0.05).

**Conclusion:**

Our results support the notion that exercise at high intensities influences inhibitory control and one’s perception of pleasure, which are linked to changes in cardiac autonomic control and cerebral hemodynamics. These findings strengthen the existence of an integrated brain-heart-body system and highlight the importance of exercise intensity in exercise-related behavior in sedentary individuals.

## Introduction

Physical inactivity is considered one of the greatest public health issues [1], [2]. Exercising regularly minimizes risk for cardiovascular and musculoskeletal health problems and improve mental health and autoimmune functioning. Even in the face of these health benefits, the lack of adherence by a large part of the population may be due to the displeasure that accompanies exercise, especially at high intensities and especially for individuals who are sedentary [3]. One possible cognitive explanation for high reports of exercise-related displeasure is that attentional focus is directed towards the physiological sensations that accompany metabolic changes induced by exercise [4]. For instance, at exercise intensities above the ventilatory threshold (VT) (i.e., a marker of the transition from aerobic to anaerobic metabolism), individuals often report increases in associative thoughts about the physiological sensations they are experiencing, as well as negative affect/displeasure [5]. At exercise intensities below the VT, positive affect is usually reported, because the same physiological sensations are minimized during “steady-states” [6]. Interestingly, a number of studies suggest that inhibitory control, defined by the ability to regulate attention and ignore irrelevant information, may mediate one’s ability to emotionally regulate sensations about his/her physiological status [7–9]. As a result, inhibitory control may be an important neurobiological mechanism that contributes to physical activity adherence [10, 11]. In other words, inhibitory control may help to regulate an exerciser’s attention to physiological changes (i.e., his/her interoception) at different intensities [12], thus regulating the degree to which pleasure or displeasure is perceived, which ultimately has an impact on future exercise behavior [13, 14].

Increases in exercise intensity influence the amount of interoceptive stimuli coming from the heart due to autonomic changes such as vagal withdrawal and sympathetic activation [15]. One theoretical model of visceral and neural integration suggests that visceral feedback (e.g., heart rate variability, HRV) interacts with networks of brain regions that support emotion processing [16]. Specifically, increased exercise intensity leads to hyperactivity of the amygdala, an area that has *bottom-up* projections to the prefrontal cortex (PFC), which supports inhibitory control [10, 13]. Exercise intensity also affects *top-down* processing, such that inhibitory control feeds back to modulate emotion. Thus, HRV may be used as a cue to generate changes in interoceptive processing during exercise, with inhibitory control tightly regulating when these interoceptive cues are ignored [12, 17, 18]. This interactive system has been explained by a neural model where the PFC actively inhibits visceral interoceptive responses (from HRV) that activate the amygdala [19]. Separate work indicates that PFC oxygenation also changes with exercise intensity: it increases when exercising at low and moderate intensities and decreases at elevated intensities [20]. This exercise-related hypofrontality impacts inhibitory control and emotional regulation and thus may have effects on perceived pleasure and exercise tolerance [21, 22]. However, the precise nature of this brain-heart-body interaction remains unclear, especially with respect to changes in exercise intensity.

In this study, we investigated the effects of exercise intensity on inhibitory control, perceived pleasure, interoception (associative and dissociative thoughts), cardiac autonomic control, and oxygenation of the PFC. Based on the extant literature, we maintained two predictions: (I) intensities above the VT will have a deleterious effect on inhibitory control and oxygenation in the PFC, accompanied by feelings of displeasure and increases in sympathetic activity; and (II) decreases in pleasure will be related to declines in inhibitory control and oxygenation of the PFC, as well as autonomic activity.

## Method

### Subjects

Of the forty participants that enrolled in the study, three were not available to attend all sessions. Thus, the final sample included 37 sedentary individuals (28 women; Age: 25.1 ± 4.6; Weight: 65.8 ± 13.0 kg; Height: 1.7 ± 0.1 m; BMI: 28.4 ± 3.6 kg·m^-2^; Body Fat: 23.7 ± 4.3% and 30.5 ± 2.9% for men and women, respectively; VO_2_ peak: 26.1 ± 8.9 ml·kg^-1^·min^-1^; Resting Heart Rate: 82.5 ± 22.1 bpm). Participants were mostly undergraduate students who were recruited via classroom announcements, fliers, and social media. The study was approved by the Federal University of Rio Grande do Norte ethics committee (CAAE: 30412214.5.0000.5537) and followed the procedures proposed by the Declaration of Helsinki. Participants signed informed consent forms after a researcher explained the procedures and purpose of the study. Participants were blind to the overall purpose the study and were told that we were trying to assess the physiological and psychological variables during exercise.

All participants were between 18 and 30 years of age; had body mass index between 18.5 and 29.9 kg.m^-2^; were sedentary/deconditioned; did not have joint, neurological, cardiovascular or respiratory limitations; had no medical contraindication to performing high-intensity exercise, and were not currently taking medications that could influence the outcome measures. Participants were classified as “sedentary” according to the International Physical Activity Questionnaire (IPAQ) [23] and “deconditioned” as having low aerobic fitness (peak oxygen consumption ≤ 33 ml·kg^-1^·min^-1^) [24]. All participants were instructed not to drink alcoholic or caffeinated beverages 24 hours prior to each experimental session.

### Experimental design

Participants visited the laboratory three times, with each visit separated by at least 48 hours. The first visit served as a screening and familiarization session. Participants were asked to complete the Physical Activity Readiness Questionnaire (PAR-Q), the International Physical Activity Questionnaire (IPAQ), and an anthropometric assessment. They then were familiarized with the protocol for the experimental exercise condition, which consisted of a six-minute baseline assessment during which cognitive performance, cerebral oxygenation, heart rate, respiratory exchange, and affective responses were collected. This was followed by a maximal graded exercise test (GXT) on a cycle ergometer during which the same variables were measured. On the second and third visits, participants completed the exercise and control conditions of the experiment. Condition order was randomized and counterbalanced across participants. During the exercise session, a maximum GXT was performed on a cycle ergometer while affective responses, PFC oxygenation, HRV, cognitive inhibition (Stroop test), and associative and dissociative thoughts (ADT) were assessed at every intensity level. During the control session, participants sat motionless on the cycle ergometer while the same measures were collected every two minutes for 20 minutes (to mirror the intensity levels of the exercise condition). At the beginning of each session, participants filled the profile of mood states (POMS) questionnaire [25].

### Procedures

#### Screening and familiarization session (Session 1)

During the first session, all participants completed an anthropometric evaluation conducted by a trained evaluator. Then, participants completed the PAR-Q to identify potential restrictions to exercise and the IPAQ to assess physical activity. Finally, participants underwent a GXT and were familiarized with all measures that would be collected in the subsequent sessions. If participants did not understand the scales or cognitive test, additional familiarization was offered until participants fully understood.

#### Exercise and control conditions (Sessions 2–3)

Exercise session. Participants adjusted the handlebar and seat height of the cycle ergometer according to their preferences. They were then fit with the mask for respiratory analysis, optodes of the near-infrared spectroscopy device, and a heart rate monitor. Then participants rested for six minutes, where we recorded baseline measurements. After this period, the GXT started on an electromagnetic cycle ergometer (Model CG04, Inbrasport, Porto Alegre, Brazil) with an initial load of 25 W and additions of 25 W for men and 15 W for women every two minutes. Participants were instructed to maintain a cadence of 60–70 r/min for the entire session. The criteria to terminate the test were: (1) voluntary exhaustion [26], (2) when participants could not maintain the established cadence (<5 rpm) for more than five seconds, or (3) if the 95% range of estimated maximum heart rate (220-age) was exceeded. Heart rate (HR), VO_2_, and oxygenation of the PFC were measured continuously throughout the entire session. The Stroop test was administered at rest and during the last minute of each stage of the GXT. Additionally, at the end of each stage, participants were asked to report their level of pleasure using the Feeling Scale, as well as their ADT [27].

Control session. In the control condition, participants sat motionless on the cycle ergometer for 26 minutes, and the same measurements were assessed at the same time points and frequency as in the exercise session described above. For comparison, the control condition was time-matched with the exercise condition.

#### Respiratory gas exchange

Pulmonary gas exchange was measured using a breath-by-breath respiratory analyzer (MetaLyzer 3B, Cortex, Germany). The ventilatory threshold (VT) was defined as (I) the increase of PetCO_2_ followed by a fall, (II) the decrease in ventilatory equivalent (VE/VCO_2_) followed by an increase, and (III) the rating of perceived exertion with scores between 12 and 14 on the Borg 6–20 scale [28]. The VO_2peak_ was established as the highest value averaged at 15s before exhaustion. The equipment was calibrated prior to each test according to the manufacturer's standardization using known concentrations of O_2_ (16%), CO_2_ (5%), and volume syringe (3L).

#### Inhibitory control

A computerized version of the Stroop test was presented using Testinpacs [29]. There are many different versions of the Stroop test, but most include a condition that requires participants to indicate the font color of color words (e.g., *blue* written in red ink). Inhibitory control is required to ignore the automatic response to read a word (e.g., *blue*), and instead respond to its font color (red). The current study used a Stroop test with three blocked conditions: the first two blocks were control conditions (i.e., indicate the ink color of a rectangle; indicate the color word that appears in white ink), and the last block contained incongruent trials (i.e., indicate the ink color of color words).

The index fingers of each hand remained on two buttons throughout the test, which was situated on the handlebar of the cycle ergometer. Eligible responses were displayed on the lower corners of the screen with one that was correct and one that was incorrect. Each stimulus was presented until the participant made a response.

In the first block, rectangles appeared in green, blue, black, or red in the center of the screen. Participants were instructed to press the button corresponding to the color of the rectangle. In the second block, the name of one of the aforementioned colors was displayed in white font. Participants were instructed to press the button corresponding to the word written. Finally, in the third and last block, the name of one of the four colors was displayed with a different font color of the set. Participants were instructed to press the button corresponding to the color of the font and to ignore the word written. All blocks contained 12 trials and the stimuli were presented randomly within each block. The average time to complete all three blocks of the task was approximately 45s. Average reaction time (RT) in milliseconds and the number of errors (n) committed were recorded for each block at each level of exercise intensity.

For the analysis, only the RT of the correct responses and of responses longer than 200ms were used [30]. To analyze the RT data, we subtracted the mean of the blocks 1 and 2 from the mean of the incongruent block. We only analyzed the error rates of the incongruent block.

#### Associative and dissociative thoughts

Associative and dissociative thoughts (ADT) were measured with the Attentional Focus Scale, which is commonly used to measure the percentage of attentional focus on physiological changes perceived during exercise [27, 31]. Specifically, this 11-point scale is used to measure the amount of associative and dissociative thoughts during physical exercise. Response options vary from 0% to 100% with eligible responses in 10% increments, where higher values indicate associative thinking attributed to thoughts related to interoceptive cues (i.e., breathing rhythm, muscle fatigue, heart rate, and temperature) and lower values indicate dissociative thoughts characterized as an executive process of "blocking" perceived physical sensations [32].

#### Affective assessment

Affective responses were assessed using the Feeling Scale, a validated self-report measure of perceived pleasure [33]. The 11-point scale includes ratings ranging from +5 "very good" to -5 "very bad", with zero (0) as “neutral”. Participants were instructed as follows: *"We would like you to remember an exercise that was very pleasant and associate that experience with the number +5 (upper limit)*. *Similarly*, *we would like you to remember a very unpleasant exercise and associate that experience with the number -5 (lower limit)*. *There is no right or wrong answer*, *so it takes sincerity and individuality to respond"* [34].

#### Heart rate and heart rate variability

For the evaluation of heart rate and heart rate variability (HRV), we used a heart rate monitor (RS800CX *training computer*, Polar^®^, *Finland*) accompanied by a transmitter belt (Polar WearLink^®^ W.I.N.D), positioned on the surface of the epidermis at the level of the xiphoid appendix. The data were automatically filtered using the software *Polar Precision Performance* (version 3.02.007) and were stored in text files and transferred to the software *HRV Kubios* (University of Eastern Finland).

We used the average of the last two minutes of the rest period as a baseline, and we calculated the average of each 2-minute stage of intensity for subsequent analysis. HRV data were analyzed using a frequency-domain method by applying a fast single-window Fourier transform over the R-R intervals. This allowed us to detect and correct harmonic signals in the presence of noise [35, 36]. The recorded spectral components were: low-frequency 0.04–0.15 Hz (LF); high-frequency 0.15–0.4 Hz (HF); and the LF/HF ratio. Both low- and high-frequency components were expressed in normalized units (n.u.).

#### Brain oxygenation

Prefrontal cortex oxygenation was measured using a near-infrared spectroscopy (NIRS) system (Imagent, ISS Inc., Champaign, IL, USA). Data were analyzed using HomER 2 and MATLAB (MathWorks, Inc., Milford, MA, USA) [37]. In all the experimental procedures, we calibrated the system using a solid phantom to validate the quality of the acquired signal.

Optodes were placed onto the head, over the right and the left prefrontal cortex, covering Fp1, Fpz, and Fp2 (according to the international EEG 10–20 system [38]). Each optode was paired with four light sources, with wavelengths of 690nm and 830nm, with different source-detector separations (1.5, 2.5, 3.5, and 4.5 cm). Cerebral oxygenation was continuously recorded during the 6-minute rest period and during the two experimental conditions. The NIRS device measured the absolute values of oxyhemoglobin (O_2_Hb), deoxyhemoglobin (HHb), and total hemoglobin concentrations ([HbT] = [O_2_Hb] + [HHb]).

Data from the 6-minute rest period of each session were used to define a reference baseline level (set as 0μM). The values of oxygenation shown in the following sections represent the mean values at each stage of the experimental conditions after correcting for activity in the respective baseline period.

### Statistical analysis

For the analysis, the data of the exercise was grouped into eight time points (stages): rest, first and last stages of GXT, VT, two stages below the VT (1-VT and 2-VT) and two stages above the VT (VT+1 and VT+2). For comparison, the data of the control condition was time-matched with that of the experimental condition.

A Shapiro-Wilk test was used to test the normal distribution of the data, and Levine’s test of homogeneity was used to test equality of variances. A paired t-test was used to compare the exercise and control conditions in terms of baseline mood states and the physiological and psychological measures during the last stage of the test. A two-way repeated measures ANOVA with condition (GXT vs. control) and time (eight stages) as factors were used to evaluate VO_2_, inhibitory control, affective responses, ADT, HRV (LF, HF, and LF/HF), and PFC oxygenation (ΔO_2_Hb, ΔHHb, and ΔHbT). Greenhouse-Geisser epsilon corrections were used whenever the sphericity assumption was violated. We evaluated any significant interactions using post hoc comparisons that were Bonferroni corrected. Finally, we computed Pearson correlation coefficients to explore the relationship between affective responses and each of our measures, including inhibitory control (RT and error), ADT, HRV (LF, HF, and LF/HF) and PFC oxygenation (ΔO_2_Hb, ΔHHb, and ΔHbT). These correlations were only conducted for data collected in the exercise condition. Statistical significance was set at p < 0.05 for all tests.

The achieved power was calculated a posteriori using Gpower software version 3.1.9.2 (Universität Kiel, Kiel, Germany), using the option “as in SPSS”. The analysis for ANOVA with repeated measures within-between groups interaction confirmed that all comparisons presented a power >93%, except for RT in the Stroop test (57%). In addition, the achieved power of the significant correlations was between 60% and 98%, except for the correlation between affective response and ADT at VT (49,9%) and ΔHHb at the last stage of the test (47,3%).

## Results

Table 1 depicts the measures that showed a change between baseline and the final stage of exercise: Affective responses, HRV (HF, LF, LF/HF), and inhibitory control (accuracy on incongruent Stroop items) were all lower following exercise. Physiological demand (VO_2_ and HR_max_), PFC oxygenation (ΔO_2_Hb, ΔHHb, ΔHbT), and attentional focus (ADT) were all higher following exercise. Exercise did not influence the RT of the Stroop test. Baseline overall mood state and the six domains that compose POMS (tension, depression, hostility, vigor, fatigue, and confusion) did not differ between conditions (*t*_(36)_ = 0.86; *p* = 0.40).

**Table 1:** Comparison between maximum exercise intensity and rest on inhibitory control, physiological, and psychological responses in young sedentary adults (n = 37).

Task	Measure	Control	Exercise	*t*_*(36)*_	*p*
Respiratory Gas Exchange	VO_2peak_ (ml·kg^-1^·min^-1^)	4.5 ± 0.6	24.8 ± 4.4	-29.11	<0.001
Exertion	RPE	-	18.6 ± 1.6	-	-
Inhibitory Control	ΔRT (ms)	305.2 ± 138.2	322.2 ± 261.7	-0.38	= 0.704
	Errors (n)	0.3 ± 0.6	1.7 ± 1.2	-7.65	<0.001
Interoception	ADT	20.3 ± 13.8	87.6 ± 10.9	-21.43	<0.001
Affective	Feeling Scale	3.7 ± 1.7	-3.9 ± 1.4	19.49	<0.001
Heart Rate	HR_max_ (bpm)	85 ± 12	171.5 ± 12.2	-35.67	<0.001
	LF_log_ (n.u.)^†^	1.8 (0.14–0.16)	1.9 (0.03–0.10)	?	<0.001
	HF_log_ (n.u.)	1.4 ± 0.3	1.2 ± 0.3	3.39	= 0.001
	LF/HF_log_	0.5 ± 0.4	0.7 ± 0.3	-3.71	= 0.001
PFC Oxygenation	ΔO_2_Hb (μM)	1.1 ± 1.4	9.4 ± 5.7	-8.43	<0.001
	ΔHHb (μM)	-0.2 ± 0.5	0.5 ± 1.7	-2.41	= 0.021
	ΔHbT (μM)	0.8 ± 1.5	10 ± 6	8.69	<0.001

**Note:** data expressed as mean ± standard deviation; VO_2peak_ = peak oxygen consumption; HR_max_ = maximum heart rate; ΔRT = reaction time difference between incongruent and congruent Stroop trials; ADT = associative/dissociative thoughts; RPE = Ratings of perceived exertion; LF = low-frequency; HF = high-frequency; n.u = normalized units; LF/HF = low-frequency/high-frequency ratio; O_2_Hb = oxy-hemoglobin; HHb = deoxy-hemoglobin; HbT = total hemoglobin; log = logarithmic transformation

† = values expressed in median and interquartile range.

### Inhibitory control

**Fig 1:**
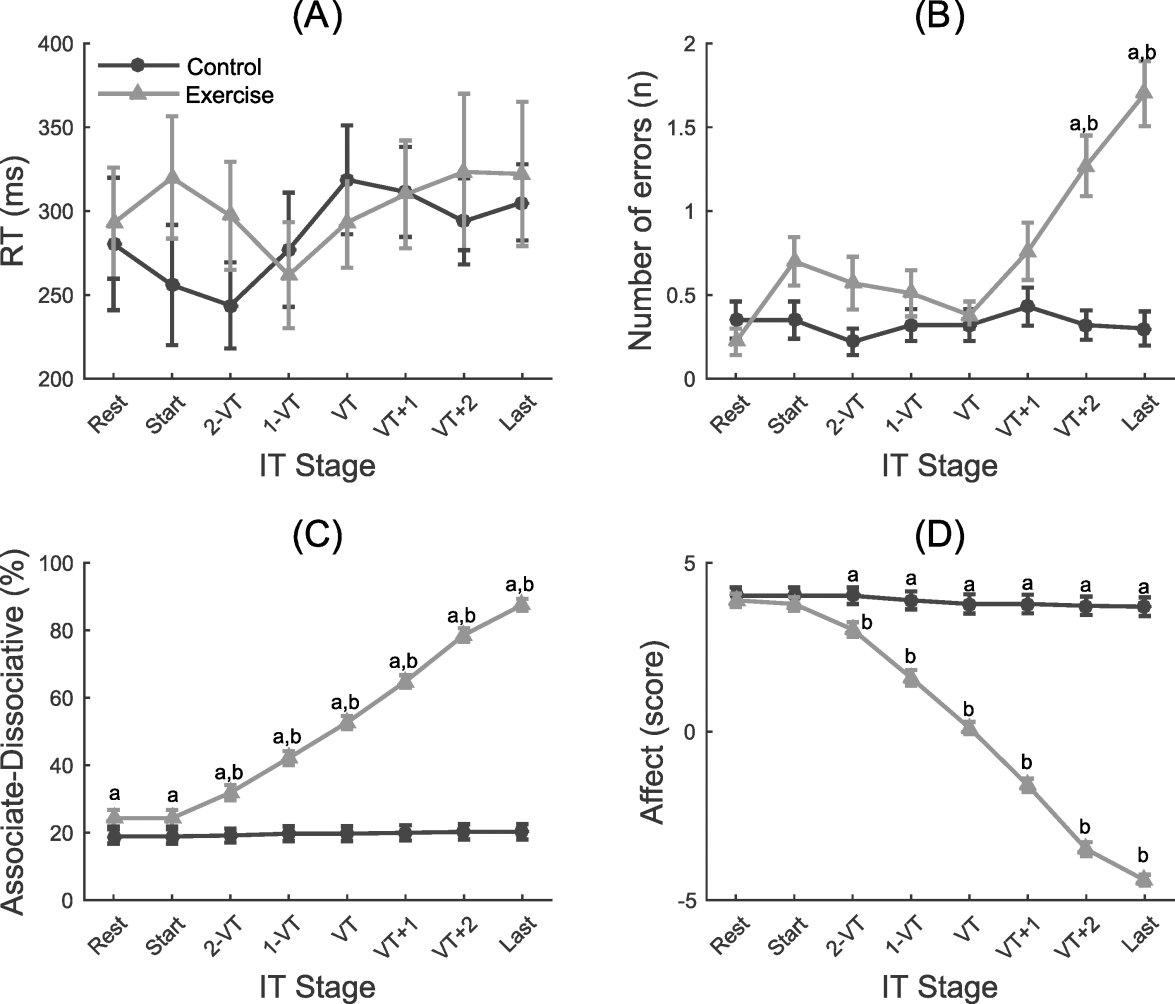
Measures of inhibitory control and emotion perception at rest and at different exercise intensities. RT = reaction time (A) and the number of errors (B) of the Stroop test, associative/dissociative thoughts (C), and affective responses on the Feeling Scale (D) for the exercise and control conditions. a = significant difference between conditions; b = significant difference from Rest; IT = incremental test; VT = ventilatory threshold.

A two-way repeated measures ANOVA revealed a significant time x condition interaction (*F*_(32.55; 121.76)_ = 9.62; *p*<0.001) for the number of errors committed. Follow-up analyses indicated higher error rates for the exercise condition at VT+2 and last stage compared to baseline (*ps*<0.001) (marked with b in Fig 1B). The exercise conditions also had higher error rates than the control condition at VT+2 and the last stage (*ps*<0.001) (marked with “a” in Fig 1B). We did not find significant effects of time (*F*_(5.14; 185.09)_ = 0.76; *p* = 0.58), condition (*F*_(1; 36)_ = 0.52; *p* = 0.47), or time x condition interaction (*F*_(5.4; 194.3)_ = 1.5; *p* = 0.17) for the RT measure of Stroop performance (Fig 1A).

### Attentional focus

There was a significant time x condition interaction (*F*_(2.28; 82.13)_ = 203.34; *p*<0.001), which was higher (i.e., more associative) at 2-VT, 1-VT, VT, VT+1, VT+2 and last stages of the exercise condition compared baseline (*ps*<0.001) (marked with b in Fig 1C). Associative thoughts were also higher for the exercise condition compared to the control condition on all stages of exercise after baseline (*ps*<0.001) (marked with “a” in Fig 1C).

### Affective responses

There was a significant time x condition interaction for self-reported responses on the feeling scale (*F*_(2.97; 106.81)_ = 324.25; *p*<0.001). In the exercise condition, affect was lower (more negative/displeasure) at 2-VT, 1-VT, VT, VT+1, VT+2 and last stages of the exercise compared to baseline (*ps*<0.001) (marked with b in Fig 1D). Affect was also lower at these same stages for the exercise condition relative to the control condition (*ps*<0.001) (marked with “a” in Fig 1D).

### HRV

**Fig 2:**
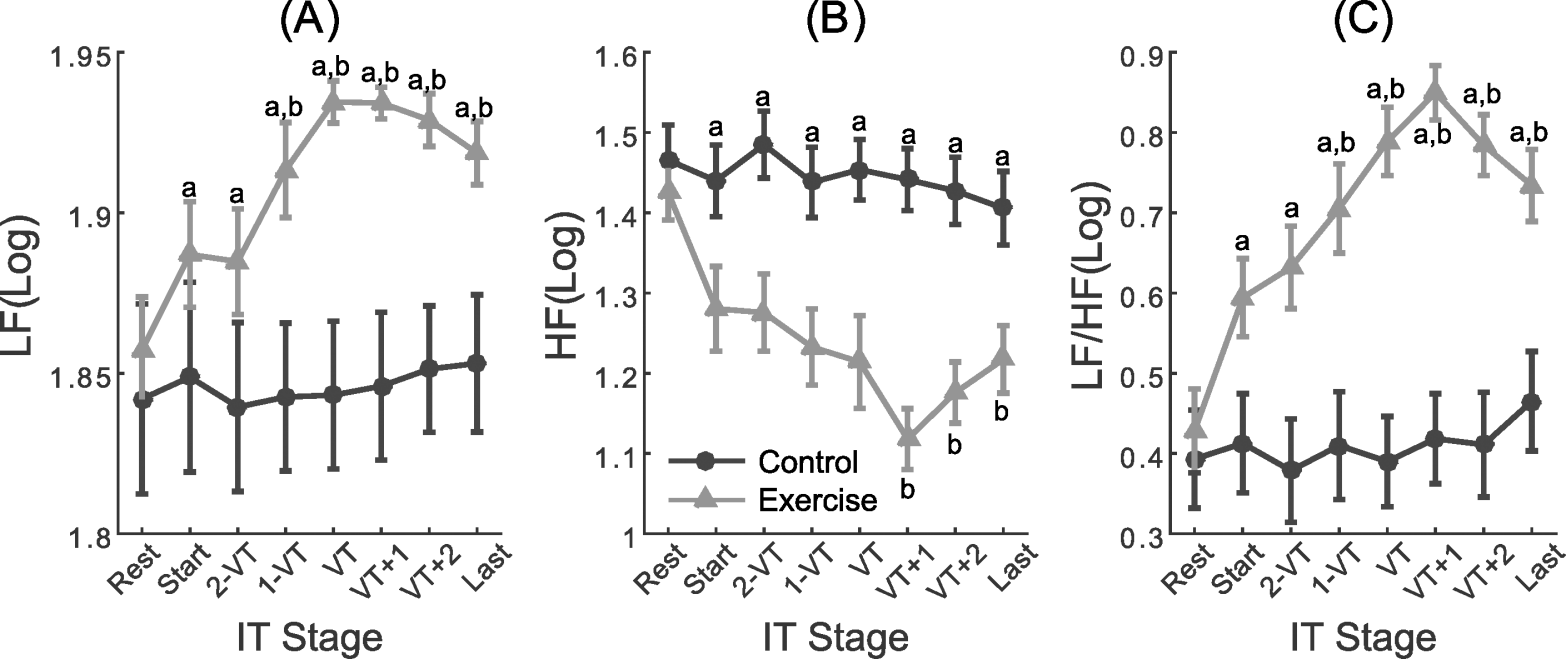
Measures of heart rate variability at rest and at different exercise intensities. Low-frequency (LF; panel A), high-frequency (HF; panel B), and low-frequency/high-frequency ratio (LF/HF; panel C). a = significant difference between conditions; b = significant difference from Rest. IT = incremental test; VT = ventilatory threshold.

There was a significant time x condition interaction for the LF index of HRV (*F*_(4.25; 153.0)_ = 3.37; *p* = 0.01). Follow-up analyses indicate that LF HRV was higher for the exercise condition compared to the control condition from the first to the last stage of exercise (*ps*<0.01) (marked with “a” in Fig 2A). Relative to baseline values, LF HRV was also higher at 1-VT, VT, VT+1, VT+2 and the last stage (*ps*<0.005) (marked with b in Fig 2A).

In addition, there was a significant time x condition interaction for the HF index of HRV (*F*_(4.9; 174.7)_ = 3.50; *p* = 0.005), which was lower during all stages of the GXT compared to control condition (*ps*<0.03) (marked with “a” in Fig 2B). HF HRV was also lower than rest in the exercise condition at VT+1, VT+2 and last stages (*ps*<0.01) (marked with b in Fig 2B).

Similarly, there was also a significant time x condition interaction for LF/HF ratio of HRV (*F*_(4.56; 164.19)_ = 5.52 *p*<0.001). The ratio was higher during all stages of the GXT compared to the control condition (*ps*<0.02) (marked with “a” in Fig 2C). It was lower than rest at VT+1, VT+2 and last stages (*ps*<0.05) (marked with b in Fig 2C).

### PFC hemodynamics

**Fig 3:**
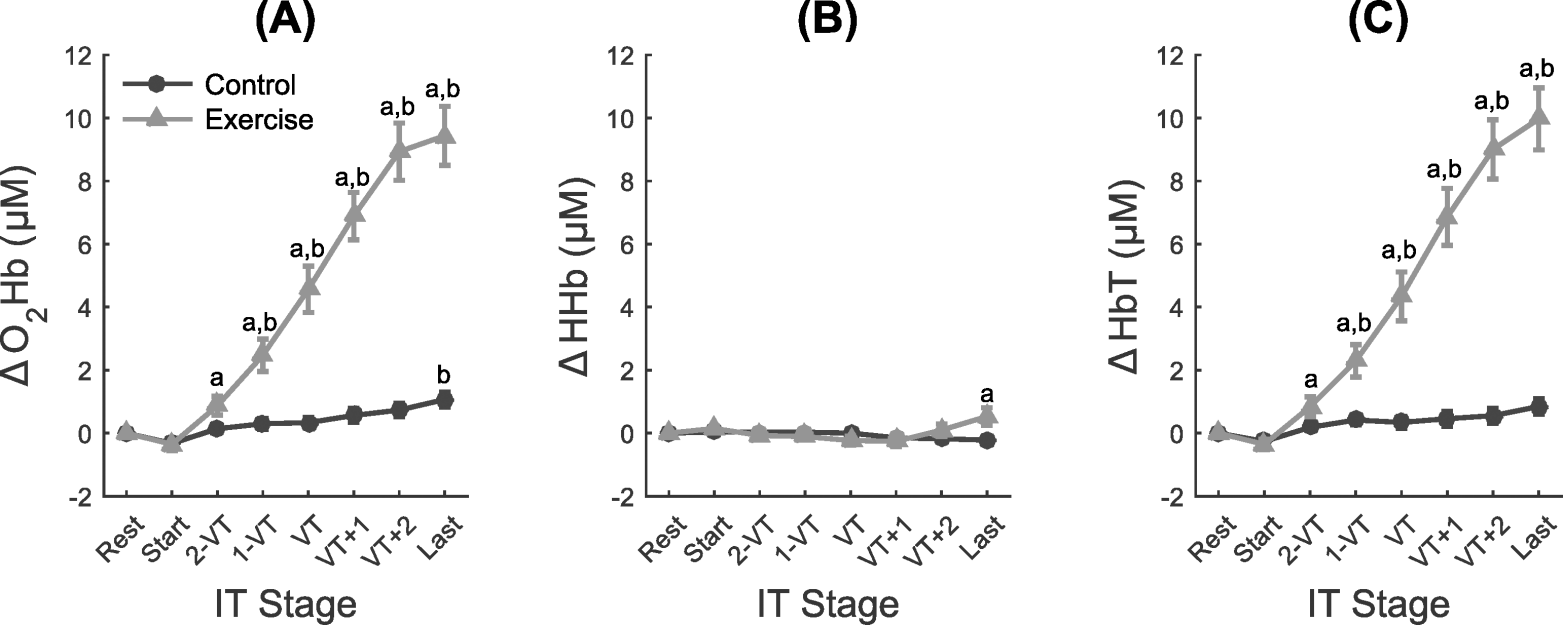
Measures of the prefrontal cortex hemodynamics at rest and at different exercise intensities relative to baseline. Oxy-hemoglobin (O_2_Hb; panel A), deoxyhemoglobin (HHb; panel B), and total hemoglobin (HbT; panel C). a = significant difference between conditions; b = significant difference from Rest. Δ—Delta percentage of variation; IT = incremental test.

There was a significant time x condition interaction on both O_2_Hb (*F*_(1.9; 67.3)_ = 55.91; *p*<0.001) and HbT (*F*_(1.9; 68.1)_ = 54.62; *p*<0.001), which were higher in the exercise condition compared to the control condition for 2-VT to the last stage of exercise (*ps*<0.05) (marked with a in Fig 3A and 3C). Both hemodynamic responses were also different than rest in the exercise condition for 2-VT to the last stage of exercise (*ps*≤0.002) (marked with b in Fig 3A and 3C). In addition, in the control condition, O_2_Hb was higher at the end of the experiment compared to baseline (*p* = 0.002) (marked with b on the blue line in Fig 3A).

For HHb, there was also a significant time x condition interaction (*F*_(1.9; 68.9)_ = 5.64; *p* = 0.006). Follow-up analyses indicated that the HHb was higher for the exercise condition compared to the control condition at last stage of the exercise (*p* = 0.02) (Fig 3B).

### Correlations between affective responses, ADT and physiological measures

Table 2 displays the correlation coefficients between affective responses and each of the other measures at each stage of intensity of the exercise condition. Significant correlations were found between affective responses and VO_2_, ADT, and HHb, particularly at the end (at high intensities) of the GXT.

**Table 2:** Correlations between affective responses and oxygen uptake, inhibitory control, associative/dissociative thoughts, heart rate variability, and prefrontal cortex oxygenation during each stage of the incremental exercise (n = 37).

Stage	Affect	VO_2_	RT	Error	ADT	LF	HF	LF/HF	O_2_Hb	HHb	HbT
Start	-	-0.05	-0.01	0.10	-0.24	0.23	-0.20	0.24	-0.14	-0.04	-0.19
2-VT	-	-0.22	-0.08	0.27	-0.46^*^	0.11	-0.12	0.11	-0.17	-0.06	-0.19
1-VT	-	-0.36^*^	-0.13	0.12	-0.38^*^	0.10	-0.06	0.15	-0.12	0.01	-0.14
VT	-	-0.08	0.07	0.04	-0.32^*^	-0.04	0.01	0.08	-0.18	-0.07	-0.19
VT+1	-	-0.07	-0.19	-0.14	-0.40^*^	-0.37^*^	0.13	-0.22	-0.17	-0.31	-0.22
VT+2	-	-0.04	-0.01	0.05	-0.55^**^	-0.02	-0.07	-0.01	0.20	-0.38^*^	0.11
Last	-	0.02	0.08	-0.13	-0.60^**^	-0.05	0.02	-0.02	0.23	-0.31^*^	0.13

VO_2_ = oxygen consumption; RT = reaction time and number of errors in the Stroop test; ADT = associative and dissociative thoughts; LF = low-frequency, HF = high-frequency, and LF/HF = low-frequency/high-frequency ratio of the HRV; O_2_Hb = Oxy-hemoglobin, HHb = deoxy-hemoglobin, and HbT = total hemoglobin of the prefrontal cortex; VT = ventilatory threshold.

*p<0.05.

**p<0.001.

## Discussion

This study revealed several interesting findings. (I) Intensities below and at the VT led to neutral feelings of pleasure and preservation of inhibitory control, while intensities above the VT resuled in displeasure and declined inhibitory control. (II) Increased intensity led to a decrease of the feeling of pleasure, with increases in associative attention. (III) The feeling of displeasure, PFC oxygenation, and sympathetic heart rate activity (LF HRV) all increased with exercise intensity.

Exercising has widely been shown to provoke systemic physiological adjustments to maintain required workload [39]. This integrated homeostatic regulation has been widely demonstrated through elevated heart rate, oxygen consumption, breathing rate, muscle recruitment, and lactate production [40], as well as altered perceptual responses of associative thoughts and affect [4]. In addition, recent studies have shown that brain function and cognitive performance are also influenced by increased exercise intensity [40, 41]. In Table 1, we showed that the effects of incremental graded exercise promoted substantial physiological, emotional, and cognitive changes, including increases in cardiovascular responses, PFC oxygenation, and associative thoughts, and decreases in pleasure and inhibitory control. Thus, our findings suggest that exercise intensity modulates physiological, perceptual, and cognitive functions. Changing exercise intensity offers an interesting approach to study the system in an integrative manner and to better understand the mechanisms contributing to physical activity adherence.

Consistent with prior work, the current findings suggest that the brain acts as an integrator of interoceptive stimuli via feedback pathways from peripheral organs that respond to the physiological demand imposed by exercise [5, 42]. It has been suggested that inhibitory control, serviced by the PFC, plays a key role in interpreting this visceral information (i.e., bottom-up) by regulating (or inhibiting) negative emotional experiences [9], which are usually signaled in subcortical structures such as the amygdala [19]. Our data show that below and at the VT, participants reported neutral feelings and preserved inhibitory control (i.e., number of errors and reaction time on the Stroop task did not differ between the exercise and control conditions). This cognitive regulation of pleasure may have occurred due to a regulatory mechanism supported by the PFC in a *top-down* fashion; by exerting control on the subcortical areas during the exposure of visceral stimuli (i.e., heart rate), this mechanism inhibits negative affective responses at lower exercise intensities [43]. However, above the VT, where the physiological demand is elevated, our data show that pleasure and performance on the inhibitory control task decreased significantly for the exercise condition compared to the control condition. Hence, it is possible that a physiological demand at these high intensities may have prevented the inhibitory control from overcoming any bottom-up signals. This claim is strengthened by the fact that participants’ lower affective ratings were associated with increases in associative attentional focus at most of the exercise intensities (Table 2). Furthermore, the relationship between affect and associative thoughts might reflect the fact that negative affect is one way that the body alerts awareness to important homeostatic disorders [6]. This attentional shift to internal physical states might act as a key factor for monitoring the system consciously and thus, preserving organic integrity under strenuous physical activity [5].

Interestingly, although the error rate increased especially after VT, the Stroop interference in RT did not (difference between incongruent and congruent phases of the Stroop test). The results presented in the literature regarding the acute effect of different exercise intensities on RT and error rates are contradictory The decrease in accuracy found in the present study is in line with a number of studies [41, 44–46]. On the other hand, Stroop interference in terms of RT did not change throughout the session, which also corroborates previous studies [44, 45]. Nonetheless, meta-analyses have shown a small increase in Stroop RT at high exercise intensities [47, 48]. One possible explanation for the discrepancy across studies could be related to the level of physical activity, fitness, type of exercise test, cognitive test type, and duration used in each study. Moreover, in cognitive tests that involve a choice between two or more response options, there is usually a trade-off between accuracy and RT. Therefore, it is likely that in the present study, participants chose to answer quicker despite the risk of committing mistakes. This trade-off would explain why error rates increased while RT did not.

A certain amount of evidence shows that the intensity of the exercise provokes an inverted "U" pattern on the PFC hemodynamics [20]. Notwithstanding, in the present study PFC oxygenation (i.e. oxyhemoglobin and total hemoglobin) increased consistently until exhaustion. Although this result seems to be surprising, recent studies have reported similar results [49, 50]. In a systematic review, Rooks et al.[20] showed that sedentary untrained and trained individuals exhibit different patterns of PFC oxygenation at different exercise intensities: trained individuals displayed an increase in oxyhemoglobin and total hemoglobin as exercise intensity increased from low to very hard, while untrained individuals showed an initial increase followed by a decrease at hard intensities. Astonishingly, the sedentary individuals of the present study displayed a similar oxygenation pattern to trained individuals in that review [20]. The reason for these differences is not clear and further research on this issue is warranted. Possible reasons that might influence the hemodynamic responses during incremental exercise could be related to differences in age, fitness level, optode placement, NIRS device used, and data processing decisions. It is important to highlight, however, that a recent theoretical model proposes that PFC may regulate the ability to tolerate high levels of physical exertion and influence the termination of exercise [13]. Additionally, Damasio [51–53] suggests that homeostatic disturbances, such as those caused by exercise, might induce a conflict between the interoceptive responses and cognitive states, which are both reliant on prefrontal regulatory functioning and are supported by *bottom-up* and *top-down* processing. Both propositions seem to fit well with our results, which showed that PFC oxygenation increased throughout the incremental exercise. Likely, this hemodynamic PFC pattern during exercise may have stifled the cognitive resources needed to manage the homeostatic imbalance (e.g., high heat accumulation, heart rate, blood pressure, blood lactate, decreased energy stores, etc.), resulting in displeasure/discomfort which may discourage an individual to continue exercising.

A recent theoretical model proposes that PFC may regulate the ability to endure high levels of physical exertion and influence when exercise fatigue occurs [13]. Additionally, Damasio suggests that homeostatic disturbances, such as those caused by exercise, might induce a conflict between the interoceptive responses and cognitive states, which are both reliant on prefrontal regulatory functioning and are supported by *bottom-up* and *top-down* processing [51, 52]. In such cases, the feeling of displeasure could be an important factor for exercise intensity tolerance. This claim fits well with our results, which show that PFC oxygenation increased throughout the incremental exercise. We believe that this hemodynamic PFC pattern during exercise may have stifled cognitive resources needed to manage the homeostatic imbalance (e.g., displeasure).

Additionally, the autonomic nervous system has been shown to regulate the exercising body, since adjusting heart rate and blood pressure is critical to providing oxygenated blood to active body tissue (e.g., muscles). It is important to highlight that this regulation is performed unconsciously by the brain stem and subcortical areas [52]. In this way, heart rate variability has become a widely-used tool to assess the changes in autonomic regulation. For example, the increased parasympathetic modulation at rest and during exercise is indexed by elevated HRV, making HRV an important marker of endurance capacity and cardiovascular health [54]. On the other hand, an increased sympathetic activity that is usually measured by decreased HRV has been associated with fatigue and exercise intolerance [55]. In this study, the HF/LF ratio and LF components of HRV were related to displeasure at the highest exercising intensity, just before exhaustion. Interestingly, these two markers are typically related to an increased sympathetic activity. Hence, the patterns we observed across these parameters suggests how the system might integrate unconscious (e.g., autonomic regulation) and conscious regulators of metabolic demand (e.g., perceived pleasure) to avoid organic damage to the system. In which, negative affect (e.g. displeasure and/or discomfort) may be the main body’ symptom bringing to awareness the homeostatic disorders [6, 14, 56] whereas persistent exercise at elevated perceived negative affect (i.e. fatigued) may deteriorate cognitive and motor functioning, being both extremely dangerous for the individuals' integrity [5, 57, 58] Moreover, PFC, which is mainly known as a cognitive brain region, has also been shown to play an important role in autonomic control, as it often processes endogenous and exogenous cues [59]. Furthermore, some work suggests that HR is linked to PFC functioning via feedback of the cardiac conditions [15]. We believe that the changes in PFC oxygenation, alongside increased sympathetic activity and increased displeasure, are critical modulators of exercise tolerance and behavior during high-intensity exercise.

We conclude that exercising at low and moderate intensities may be used to encourage future exercise adherence in sedentary individuals since feelings of pleasure remain positive and inhibitory control is preserved at these lower intensities. On the other hand, exercising at high intensities leads to displeasure and induces a temporary decline in inhibitory control, which may be less likely to promote maintenance in exercise programs. In addition, displeasure at high-intensity exercise is related to cardiac autonomic regulation, brain hemodynamics, and body consciousness, which suggests an integrative system based on affective perception to regulate the side effects that come with changes in exercise intensity. This perspective may be important when proposing practical physical interventions, since enhancing positive affect and regulatory processing while exercising may increase the likelihood of exercise-related behavior in sedentary individuals.

## Supporting information

S1 Fig Measures of inhibitory control and emotion perception at rest and at different exercise intensities. (TIFF)

S2 Fig Measures of heart rate variability at rest and at different exercise intensities. (TIFF)

S3 Fig Measures of the prefrontal cortex hemodynamics at rest and at different exercise intensities relative to baseline. (TIFF)

S1 Table Comparison between maximum exercise intensity and rest on inhibitory control, physiological, and psychological responses in young sedentary adults (n = 37). (PDF)

S2 Table Correlations between affective responses and oxygen uptake, inhibitory control, associative/dissociative thoughts, heart rate variability, and prefrontal cortex oxygenation during each stage of the incremental exercise (n = 37). (PDF)

S1 Data Characteristics of the sample and individual responses to VO_2_; Stroop test: RT and Error; ADT; Affective; HRV: LF, HF and LF/HF; Brain oxygenation: O_2_HB, HHb and HbT. (XLSX)
