# Supplementary figures and images for: Affect during incremental exercise: The role of inhibitory cognition, autonomic cardiac function, and cerebral oxygenation
Source: PLoS One. 2017 Nov 1;12(11):e0186926. doi: 10.1371/journal.pone.0186926 (PMC5665513; doi:10.1371/journal.pone.0186926)

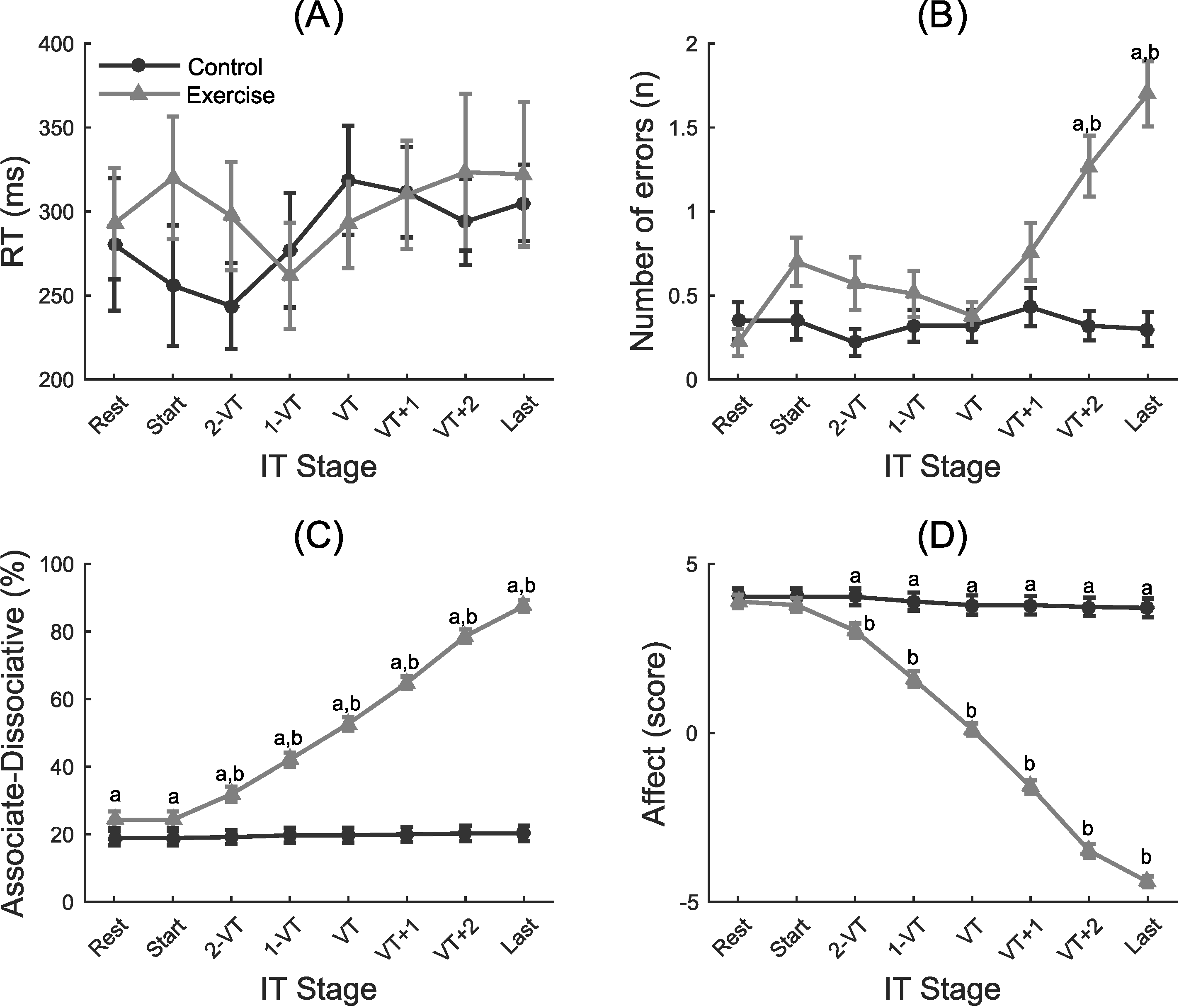

Supplement: S1 Fig — (TIFF) [file pone.0186926.s001.tiff]

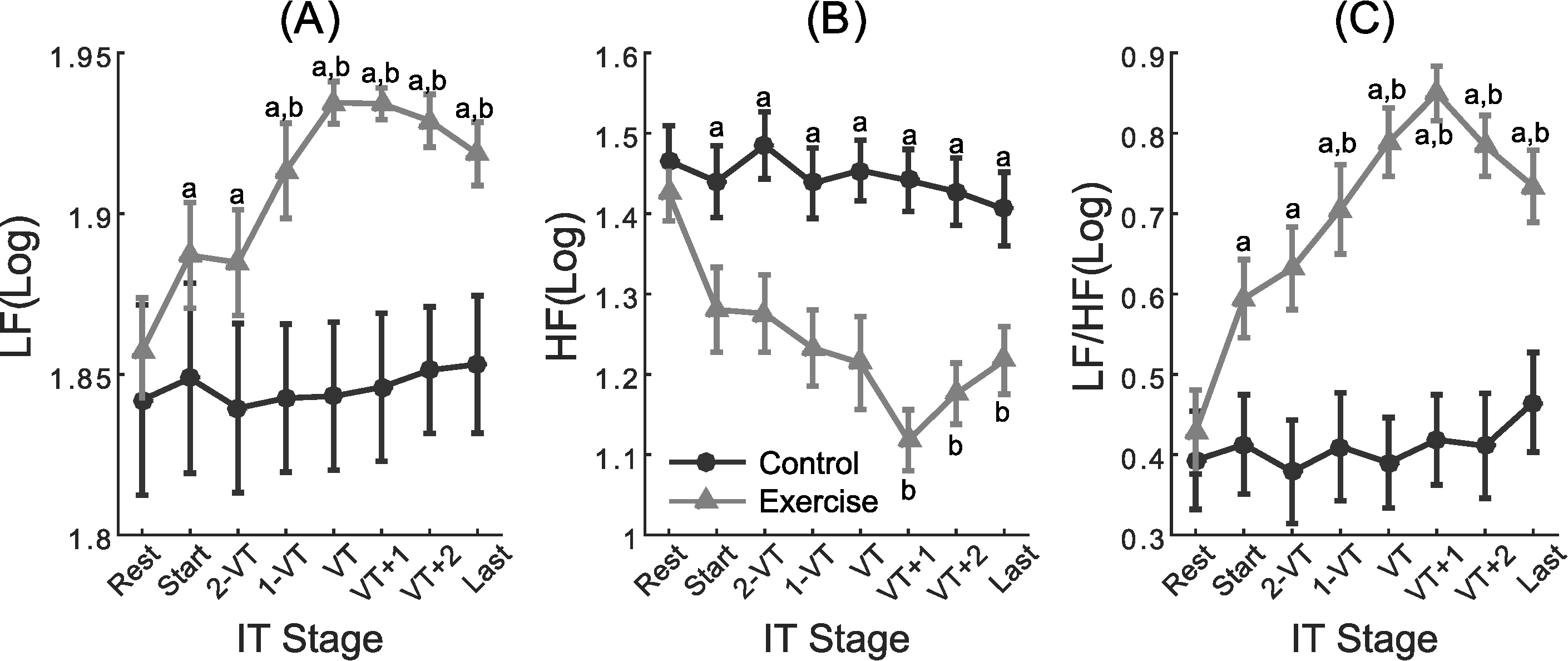

Supplement: S2 Fig — (TIFF) [file pone.0186926.s002.tiff]

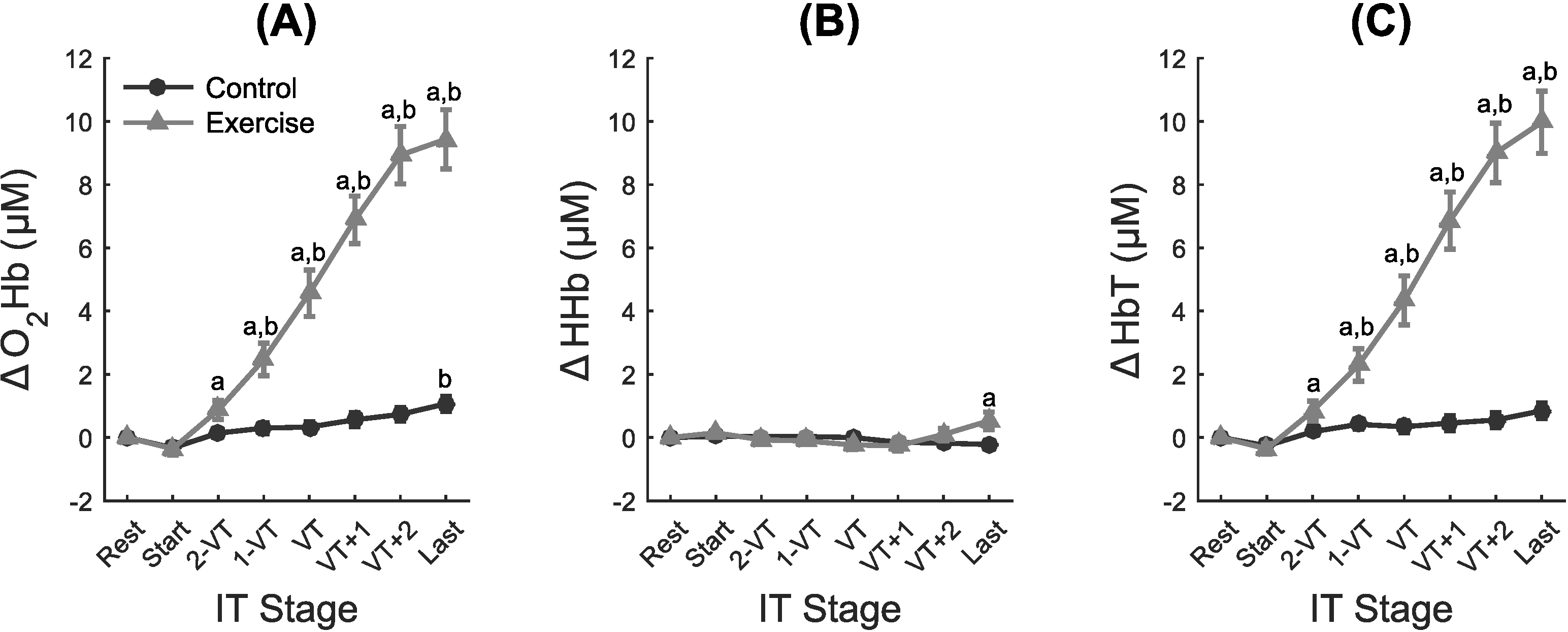

Supplement: S3 Fig — (TIFF) [file pone.0186926.s003.tiff]
