# Supplementary material for: Affect during incremental exercise: The role of inhibitory cognition, autonomic cardiac function, and cerebral oxygenation
Source: PLoS One. 2017 Nov 1;12(11):e0186926. doi: 10.1371/journal.pone.0186926 (PMC5665513; doi:10.1371/journal.pone.0186926)
Supplement: S1 Table — (PDF) [file pone.0186926.s004.pdf]

Task	Measure	Control	Exercise	$t_{(36)}$	p
Respiratory Gas Exchange	VO_{2peak} (ml·kg^{-1}·min^{-1})	4.5 ± 0.6	24.8 ± 4.4	-29.11	<0.001
Exertion	RPE	-	18.6 ± 1.6	-	-
Inhibitory Control	ΔRT (ms)	305.2 ± 138.2	322.2 ± 261.7	-0.38	=0.704
	Errors (n)	0.3 ± 0.6	1.7 ± 1.2	-7.65	<0.001
Interoception	ADT	20.3 ± 13.8	87.6 ± 10.9	-21.43	<0.001
Affective	Feeling Scale	3.7 ± 1.7	-3.9 ± 1.4	19.49	<0.001
Heart Rate	HR_{max} (bpm)	85 ± 12	171.5 ± 12.2	-35.67	<0.001
	LF_{log} (n.u.)†	1.8 (0.14 – 0.16)	1.9 (0.03 – 0.10)	?	<0.001
	HF_{log} (n.u.)	1.4 ± 0.3	1.2 ± 0.3	3.39	=0.001
	LF/HF_{log}	0.5 ± 0.4	0.7 ± 0.3	-3.71	=0.001
PFC Oxygenation	ΔO_2Hb (µM)	1.1 ± 1.4	9.4 ± 5.7	-8.43	<0.001
	ΔHHb (µM)	-0.2 ± 0.5	0.5 ± 1.7	-2.41	=0.021
	ΔHbT (µM)	0.8 ± 1.5	10 ± 6	8.69	<0.001
