# Supplementary material for: Affect during incremental exercise: The role of inhibitory cognition, autonomic cardiac function, and cerebral oxygenation
Source: PLoS One. 2017 Nov 1;12(11):e0186926. doi: 10.1371/journal.pone.0186926 (PMC5665513; doi:10.1371/journal.pone.0186926)
Supplement: S2 Table — (PDF) [file pone.0186926.s005.pdf]

Stage	Affect	VO_2	RT	Error	ADT	LF	HF	LF/HF	O_2Hb	HHb	HbT
Start	-	-0.05	-0.01	0.10	-0.24	0.23	-0.20	0.24	-0.14	-0.04	-0.19
2-VT	-	-0.22	-0.08	0.27	**-0.46***	0.11	-0.12	0.11	-0.17	-0.06	-0.19
1-VT	-	**-0.36***	-0.13	0.12	**-0.38***	0.10	-0.06	0.15	-0.12	0.01	-0.14
VT	-	-0.08	0.07	0.04	**-0.32***	-0.04	0.01	0.08	-0.18	-0.07	-0.19
VT+1	-	-0.07	-0.19	-0.14	**-0.40***	**-0.37***	0.13	-0.22	-0.17	-0.31	-0.22
VT+2	-	-0.04	-0.01	0.05	**-0.55****	-0.02	-0.07	-0.01	0.20	**-0.38***	0.11
Last	-	0.02	0.08	-0.13	**-0.60****	-0.05	0.02	-0.02	0.23	**-0.31***	0.13
